# Cortisol Is Not Associated with Telomere Shortening or Chromosomal Instability in Human Lymphocytes Cultured under Low and High Folate Conditions

**DOI:** 10.1371/journal.pone.0119367

**Published:** 2015-03-06

**Authors:** Caroline Bull, Helen Christensen, Michael Fenech

**Affiliations:** 1 Nutritional Genomics and DNA Damage Diagnostics Laboratory, CSIRO Animal, Food and Health Sciences, Adelaide, South Australia, Australia; 2 Department of Microbiology & Immunology, School of Molecular & Biomedical Science, University of Adelaide, Adelaide, South Australia, Australia; 3 Black Dog Institute, Prince of Wales Hospital, Randwick, New South Wales, Australia; University of Newcastle, UNITED KINGDOM

## Abstract

Chronic psychological stress and nutritional deficiencies are factors that impact negatively on human health and disease risk. Chronic stress has been associated with accelerated leukocyte telomere shortening in numerous cohorts, however, a mechanistic link has proven elusive. This study tested the hypotheses that chronic exposure to the stress hormone, cortisol, causes telomere shortening and chromosome instability (CIN) *in vitro*, and that these effects would be further exacerbated by folate (vitamin B9) deficiency. Primary human lymphocytes were maintained *in vitro *for 12 days in medium containing either 25 nM folic acid (FA(low)) or 100 nM FA (FA(high)), together with either 0, 400, 1000 or 3500 nM cortisol. The interactive effects of cortisol and FA were examined by comparing telomere length (TL), biomarkers of DNA damage, and cytostasis. At day 12 TL was 5-17% longer in lymphocytes cultured in FA(low) conditions (mean ± SD;10.2% ± 1.6), compared with those in FA(high) medium (9.1% ± 1, p = 0.02). Refuting the hypothesis, TL was consistently greater in the presence of cortisol. The effect of FA deficiency on the frequency of DNA damage was significant for nucleoplasmic bridges, circular nuclei, micronuclei and nuclear buds, (p < 0.0001 – 0.001). The effect of cortisol, however, was negligible, only reaching statistical significance for the frequency of fused nuclei (p = 0.04). Cortisol was significantly associated with reduced cell division and growth and had an apparent protective effect on cell viability in the FA(low) conditions. Conclusions: Both chronic cortisol exposure, and folate deficiency, resulted in telomere elongation, however, the effect of cortisol was marginal relative to that of folate. Cortisol was not associated with increased chromosomal instability, but caused a significant reduction in cell division and growth. Together these results indicate that cortisol is not directly genotoxic and that the telomere shortening associated with increased psychological stress *in vivo* may not be explained by a direct effect of cortisol.

## Introduction

Telomeres are nucleoprotein structures which cap and protect chromosome ends, and shorten with each cell division due to the inability of DNA polymerases to completely replicate the terminal telomere sequences [1, 2]. Critically short telomeres are associated with chromosomal instability (CIN) and cellular senescence, each of which increases risk for degenerative diseases of ageing [1, 2]. The rate of telomere attrition differs between individuals due to variability in genetic, environmental, dietary and life-style factors [3–5].

Psychological stress is one factor that has been associated with accelerated telomere shortening in lymphocytes or leukocytes. Numerous studies have shown that chronically stressed individuals have significantly shorter telomeres than those with lower perceived and/or actual stress [5–7], and that stress and adversity experienced during childhood results in shortened telomeres and chromosomal damage (micronuclei) in adults [8–10]. These findings are consistent with a wealth of evidence suggesting that psychological stress impacts deleteriously on human health at the chromosomal level [11, 12]. To date, however, a clear direct or indirect mechanistic link between psychological stress and shortened telomeres has proven elusive. The stress response is mediated by the hypothalamus-pituitary-adrenal (HPA) axis, a central physiological feature of which is the release of the stress hormones, cortisol and noradrenaline [12, 13]. Regulation of these hormones is critical for activation (and timely shutdown) of the fight/flight response, whereas under chronic stress conditions these hormones may remain elevated for extended periods of time [11, 12].

Micronutrient deficiencies also have serious health consequences, including compromised telomere integrity and function, increased CIN, and adverse changes in gene transcription, potentially initiating developmental and degenerative diseases [14]. B-group vitamins are essential both for accurate replication of DNA in dividing cells, and maintenance of methylation (epigenetic) patterns [15]. Folate (vitamin B9) deficiency is a recognized genotoxic insult (of a magnitude similar to exposure to damaging doses of ionizing radiation) [16], impacting telomere length and DNA integrity via two discrete, yet interrelated, pathways. These are (i) replication stress, leading to DNA breaks and terminal chromosome deletions, and (ii) hypomethylation of repeat sequences in pericentromeric and subtelomeric regions of the chromosome [17, 18]. Recent evidence suggests that deficiency in B vitamins may also increase susceptibility to psychological distress, which may be mitigated by high-dose B vitamin supplementation [19, 20]. It is also possible that poor eating habits occur in connection with psychological stress which may lead to metabolic disorders such as obesity or deficiencies in key nutrients, including B vitamins [21, 22].

Plasma concentrations of folate and its metabolite, homocysteine (Hcy), are inversely related because folate is the methyl donor required for the metabolism of homocysteine to methionine [15]. Elevated Hcy is strongly associated with numerous morbidities, including Alzheimer’s and cardiovascular diseases [23, 24]. Psychological stress has been associated with high Hcy, most notably in older individuals [25]. High serum cortisol has also been significantly correlated *in vivo* both with low folate, and high Hcy [26, 27]. Direct intramuscular injection of cortisol, or its precursor ACTH, resulted in a 23% and 24% reduction in serum folate, respectively [28]. Both psychological stress and folate deficiency have been associated *in vivo* with increased DNA damage in the form of micronuclei (indicative of chromosome breakage due to DNA double strand breaks or chromosome malsegregation) [10, 15, 29]. Furthermore, we have shown that chronic long term folate deficiency *in vitro* leads to both dysfunctional long telomeres, and loss of telomeres due to accumulation of terminal deletions [17]. Taken together, these findings suggest the plausibility of a common biochemical link between psychological stress and folate deficiency, or the possibility of a coincidence of these two factors affecting genome integrity, that warranted detailed investigation.

Accordingly, in this study we examined human lymphocytes *in vitro* to test the hypotheses that (i) continuous exposure to the stress hormone, cortisol, induces telomere shortening and CIN, and (ii) that folate deficiency will exacerbate susceptibility to cortisol-induced CIN and telomere attrition.

## Materials and Methods

### Study design

To test the hypotheses, peripheral blood lymphocytes (PBL) from six individuals were maintained *in vitro* for 12 days in one of eight different media conditions; 4 deficient for FA (FA(low), 25 nM), and four replete for FA ((FA(high), 100 nM). Cortisol (cort) was added to corresponding ‘FA(low)’ and ‘FA(high)’ treatments to final concentrations of either 400 nM, 1000 nM or 3500 nM. These doses were selected based on preliminary pilot data. Of the cort concentrations used, 400 nM is within the physiologically normal range (as defined by the diagnostic laboratory reference range; 200–700 nmol/L), whereas serum concentrations nearing 1000 nM have been reported under certain acute, high stress or experimental conditions [30, 31]. The supraphysiological condition (3500 nM) was included for the experimental purpose of potentially observing a dose effect. FA(low) and FA(high) ‘vehicle control’ (VC) (0 nM cort) cultures were maintained with 0.1% ethanol (EtOH). In several previous studies long term cultures of at least 9–12 days duration were required to observe the DNA damaging effects of chronic folate deficiency on metabolic stress [32, 33]. Accordingly, the interactive effects of cort and FA on chromosome stability and cytostasis were examined at day 12 by comparing telomere length (TL), global DNA methylation status, cell growth, viability, and biomarkers of DNA damage.

### Culture medium

FA-free RPMI 1640 was prepared from 10x stock solution (Sigma, R1145) with milliQ water, containing 2 mg/L NaHCO_3_ (Sigma, S5761) (as per manufacturer’s instruction). Medium was supplemented with 10% foetal bovine serum (FBS) (Bovogen), (v/v) 1% penicillin/streptomycin (Sigma, P4458), and 1% sodium pyruvate (Sigma, S8636). To achieve the required final FA concentrations for FA(low) (25 nM) and FA(high) (100 nM) conditions, FA-free medium was substituted with standard RPMI 1640 (Sigma, R0883). Final [FA] was confirmed by the certified clinical biochemistry laboratory at the Institute of Medical and Veterinary Science (IMVS, South Australia), and media aliquotted into 8 separate batches prior to cort addition. A 50 μg/mL stock solution of cort (hydrocortisone, Sigma H0888) was prepared by dissolving 1 mg in 1 mL absolute ethanol (EtOH), then diluting with 19 mL complete medium (CM). Stock was added to each batch of medium to achieve final concentrations of 400, 1000 and 3500 nM cort. All cultures contained 0.1% EtOH, including a 0 μM cort condition (the ‘vehicle control’ (VC)). Media aliquots were stored at -20°C and thawed at 4°C prior to use. L-Glutamine (1% v/v) (Sigma, G7513) was added to medium immediately prior to use.

### Volunteers and sample collection

Fasted, morning venous blood samples (120–150 mL) were collected on a single occasion (between 0820 and 0930h) from 3 male and 3 female volunteers (aged 53 ± 3 years). Their baseline serum concentrations (mean ± SD) of cort (424.3 ± 169.3 nmol/L), folate (32.4 ± 5.3 nmol/L), vitamin B12 (313.3 ± 76.4 pmol/L) and plasma homocysteine (8.78 ± 0.9 μmol/L) were determined by IMVS, and were within standard reference ranges for normal, healthy individuals in Australia.

### Ethics statement

The study was approved by the Human Research Ethics Committee (HREC approval #10/01) of CSIRO Health Sciences and Nutrition, Adelaide, Australia, and written informed consent was obtained from all participants.

### Cell culture

PBL were isolated by Ficoll-Paque gradient separation (Amersham Biosciences). Cell counts (Coulter Counter; Beckman Coulter) and viability tests (trypan blue exclusion; Sigma, T8154) were conducted, and cells transferred directly into flasks containing pre-warmed treatment medium. Baseline seeding density was dependent upon total number of cells obtained, and was established at a consistent initial concentration for all flasks for each donor (0.3–0.5 x 10^6^ / mL). Phytohaemagglutinin (PHA) (Oxoid; 30 μg/mL) and interleukin-2 (IL2) (10 units/mL) (Roche Diagnostics, 11147528001) were added directly to each culture flask at day 0 to stimulate division of lymphocytes. Cell cultures for each condition were maintained in duplicate 20 mL cultures, in 25cm^2^ vented-cap flasks (Becton Dickinson), and incubated in a 37°C humidified atmosphere with 5% CO_2_. Medium was replaced at day 6, incorporating 5% conditioned (spent) medium as a source of growth factors. On days three and nine 10 mL (50%) spent medium was replaced in each culture. Cells were harvested at day 12 by centrifugation, and subcultures established for the CBMN-cyt assay (see below). Aliquots of cells were viably stored at -80°C in FBS (Bovogen, Australia) containing 10% dimethyl sulphoxide (DMSO) (Sigma, Australia).

### Telomere length measurement

Telomere length (TL) was measured in cells at G1, using the flow cytometric method described previously [4]. In brief, fixed and permeabilized cells were labelled with an 18mer FITC-conjugated peptide nucleic acid (PNA) probe complementary to the telomere repeat sequence, using kit K5327 (Dako, Denmark), and counterstained with propidium iodide (PI) to measure DNA content and identify cell cycle stage. As a reference, cells from a tetraploid line with long telomeres (cell line 1301; accession number 01051619, European Collection of Cell Cultures, UK) were included in all tubes and used to calculate the relative TL in lymphocytes in the test samples. Each sample was prepared in paired tubes. For the purpose of quantifying background fluorescence of both the sample and reference cells one tube was incubated in hybridisation mixture with the PNA, while the paired tube was incubated in hybridisation mixture only. TL and DNA content measurements were acquired using a FACSCalibur flow cytometer (Becton Dickinson) and analysed using BD CellQuestTM Pro software (v5.2). A mean FITC fluorescence value was obtained for the sample and 1301 (reference) cells in the labelled and unlabelled samples by gating specifically at G0–1 phase of the cell cycle. TL of sample cells relative to that of 1301 cells was then calculated, with correction for ploidy (DNA index) of the different cell populations. The coefficient of variation (mean ± SD) of duplicate measurements was 5.0 ± 5.7%.

### Chromosomal damage, nuclear division index and cytostasis (CBMN-cyt assay)

Chromosomal instability was determined using the cytokinesis-block micronucleus cytome (CBMN-cyt) assay, with minor modifications to the standard protocol that has been described in detail elsewhere [34]. In brief, at day 12 duplicate 500 μL sub-cultures were established, at a concentration of 0.5 x 10^6^ viable cells/mL. Cytochalasin B (CytB) (4.5 μg/ml; Sigma, Australia) was added to block cytokinesis, and cells were harvested 26h later by cytocentrifugation using a Shandon Cytospin Cytocentrifuge (Shandon Scientific, Cheshire, England). Slides were air-dried (20 min), fixed and stained using Hemacolour (Merck Chemicals, Germany). All slides were blinded, coded and scored by one person (CB), using established criteria [34, 35]. In all, 500 binucleated (BN) cells per duplicate culture (total 1000 BN cells per treatment) were scored. Cytostasis measures were determined by scoring the frequency of cells displaying mononuclear, BN, multinucleated, necrotic or apoptotic morphologies, per 500 cells. Nuclear division index (NDI) was calculated as NDI = (M1 + 2M2 + 3M ≥ 3) / *N*, where M1, M2 and M ≥ 3 represent the number of cells with 1, 2 or ≥3 nuclei, and *N* is the total number of viable cells scored (i.e. excluding necrotic and apoptotic cells).

### DNA isolation and global DNA methylation

DNA was isolated using a DNEasy blood and tissue kit (Qiagen, Cat no.69506) as per manufacturer’s instructions. Methyl-cytosine content, as a percentage of total cytosine content, was estimated using the MethylFlash Methylated DNA Quantification Kit (Colorimetric) (Epigentek, USA, Catalog No P-1034), following manufacturer’s instructions. The percentage of methylated cytosines in each sample was estimated using the formula: % 5-meC = ((sample OD − neg control OD) / S) / ((pos control OD − neg control OD) x 2 / P) x 100%, where OD is the optical density reading for each well (at 450 nm), ‘neg control’ is negative control, S is the amount of input sample DNA in ng (ie. 100), ‘pos control’ is positive control, and P is the amount of positive control (ng). The numeral 2 in the denominator is required to normalise 5-meC in the positive control to 100%, as the standard provided in the kit is only 50% methylated.

### Statistical analyses

Two-way analysis of variance (ANOVA) was used to define (i) the degree of variance in the data which is attributable to FA or cort, individually, and (ii) the variance due to the interactive impact of these two factors. Pair-wise comparison of significance was determined using Bonferroni posthoc tests, or Student’s t-test. Significance was accepted at p < 0.05. All statistical analyses were performed using Graphpad PRISM 4.0 (GraphPad Inc., San Diego, CA).

## Results

### Telomere length and global DNA methylation

**Fig 1:**
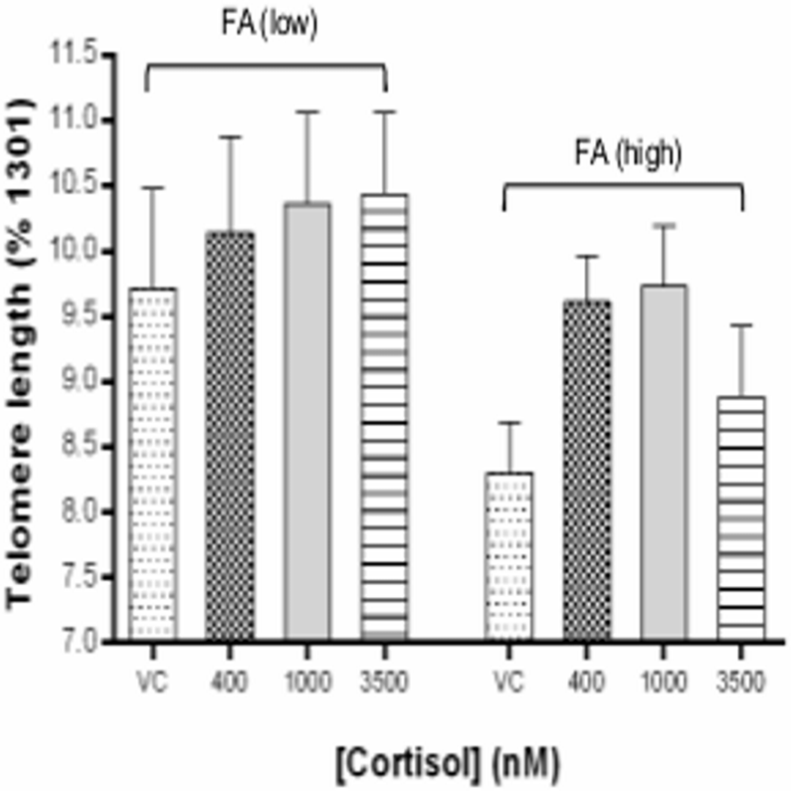
Impact of cortisol on lymphocyte telomere length (TL) following 12 days in low (25 nM) or high (100 nM) folic acid (FA) culture conditions. TL measured by flow cytometry, expressed relative to the reference standard (1301) cell line (%). Each bar represents duplicate measures from n = 6 participants (mean ± SEM; VC, vehicle control).

Telomere length (TL) of lymphocytes following 12 days of culture in FA-deficient (FA(low), 25 nM) conditions was 5–17% longer than that of cells from FA-replete (FA(high), 100 nM) conditions (Table 1, Fig. 1). Mean TL for all FA(low) cultures was 10.2% ± 1.6 (mean ± SEM), 11% greater than the mean TL of the four FA(high) cultures (9.1% ± 1.1) (t test, p = 0.02). Two-way ANOVA analysis showed [FA] was responsible for 12% of variance in TL (p = 0.02), 7% was attributable to [cort] (p = 0.3), and only 2.3% to their interaction (p = 0.7).

**Table 1:** Lymphocyte telomere length.

Cortisol concentration (nM)	25 nM FA	100 nM FA	Difference in TL in low vs high FA cultures (%)	TL P value (t test)
	TL / (% 5-meC)	TL / (% 5-meC)		
0 (VC)	9.7 ± 1.9	8.3 ± 0.9	- 16.8	0.13
	(2.5 ± 0.6)	(2.7 ± 1.6)		
400	10.1 ± 1.8	9.6 ± 0.9	- 5.2	0.52
	(2.8 ± 1.0)	(2.5 ± 1.0)		
1000	10.4 ± 1.7	9.7 ± 1.1	- 7.2	0.47
	(3.0 ± 0.7)	(2.4 ± 0.9)		
3500	10.4 ± 1.5	8.9 ± 1.3	- 16.8	0.09
	(2.2 ± 0.8)	(2.4 ± 0.5)		

Telomere length (TL, %1301) following 12 days culture in medium containing low (25 nM) or high (100 nM) folic acid (FA), in combination with 0 nM, 400 nM, 1000 nM or 3500 nM cortisol. Global methylation status (% 5-meC) is indicated in parentheses.

Mean ± SEM; duplicate measures from N = 6 participants; VC, vehicle control

Within each grouping of FA(low) or FA(high) cultures, the longest telomeres were observed in the cultures containing cort; the three longest being in the FA(low) 3500 nM cort (10.43 ± 1.5%), 1000 nM cort (10.36 ± 1.7%) and 400 nM cort (10.14 ± 1.8%), respectively. The longest TL overall (the ‘FA(low), 3500 nM cort’ condition) was 3% longer than that of cort-free FA(low) control (VC) (p = 0.48, ns), and 26% longer than the corresponding FA(high) control (VC) (p = 0.02) (Fig. 1).

One possible mechanism which has been associated with telomere elongation under folate deficient conditions is DNA hypomethylation [17, 36]. Accordingly, global methylation status of cells from each condition was determined by testing the percentage of methylated cytosine residues (%5-meC) (Table 1). Consistent with this hypothesis the culture with the longest TL (‘FA(low), 3500 nM cort’) also recorded the lowest methylation (2.2% ± 0.8). Also, consistent with expectation, mean %5-meC for FA(low) cultures (2.61 ± 0.36) was 4% lower than that of the FA(high) cultures (2.5 ± 0.13) (ns). Methylation status overall was not, however, associated with TL (pearson’s r = 0.03, p = 0.9). ANOVA analysis indicated that [cort] explained 2.8% of variance (p = 0.7), [FA] 0.4% of variance (p = 0.7), and 4% of variance being attributable to the interaction of these factors (p = 0.6, ns).

### Biomarkers of DNA damage and chromosomal stability

**Fig 2:**
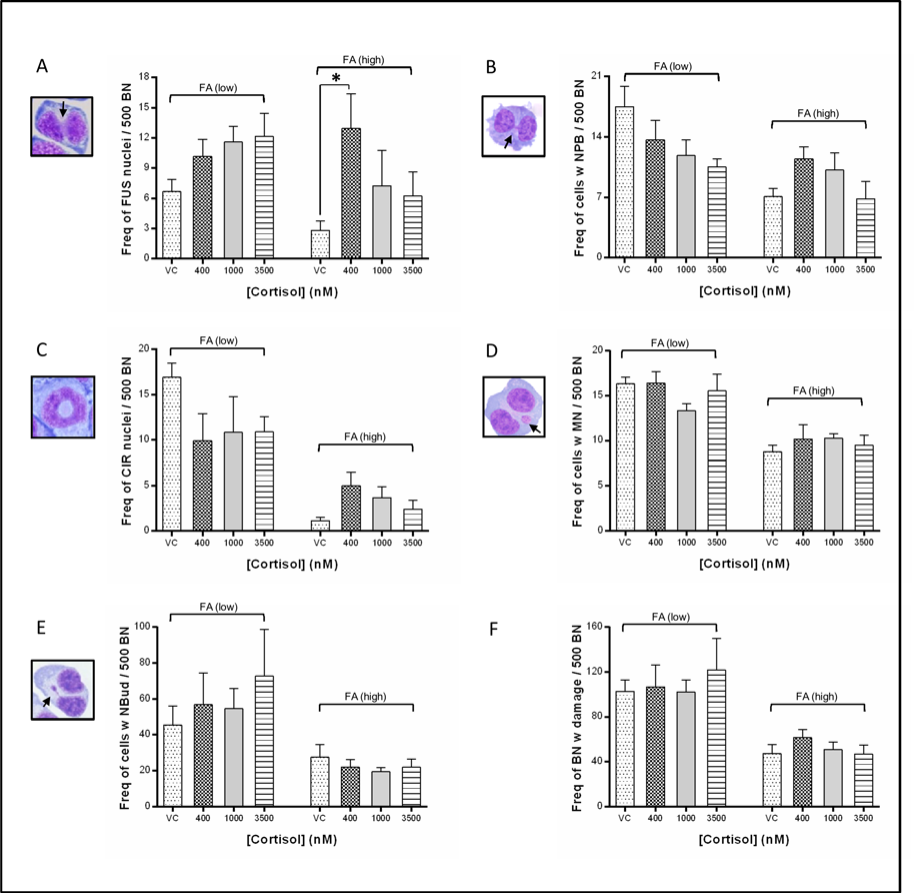
Biomarkers of DNA damage and chromosomal instability. Frequency of binucleated (BN) lymphocytes displaying one or more DNA damage biomarker (per 500 BN), following 12 days in low (25 nM) or high folic acid (FA) (100 nM) culture medium containing 0, 400, 1000 or 3500 nM cortisol: BN with (A) fused nuclei (FUS), (B) ≥1 NPB, (C) circular nuclei (CIR), (D) ≥1 MN, (E) ≥1 NBud, and (F) total frequency of BN cells containing one or more DNA damage biomarker. (mean ± SEM; 500 BN scored per duplicate slide per treatment, N = 6 participants; *represents p ≤ 0.05; VC, vehicle control).

DNA damage was determined using the CBMN-cyt assay [34]. Consistent with the known genotoxic effect of FA deficiency the frequency of binucleated (BN) cells containing a damage biomarker was significant for nucleoplasmic bridges (NPB) (p = 0.001), circular nuclei (CIR) (p < 0.0001), micronuclei (MN) (p < 0.0001), and nuclear buds (NBud) (p = 0.0005). The effect of [cort], however, was negligible, only reaching statistical significance for the frequency of fused nuclei (FUS) (p = 0.04) (Table 2, Fig. 2).

**Table 2:** Impact of folic acid and cortisol on biomarkers of chromosomal instability.

		FA	Cortisol	Interaction
		(var / p)	(var / p)	(var / p)
A	FUS	5.7%	15.4%	7.7%
		0.08	0.04	0.24
B	NPB	19.2%	8.9%	11.5%
		0.001	0.14	0.07
C	CIR	38.0%	6.7%	2.4%
		<0.0001	0.19	0.68
D	MN	53%	0.7%	4.1%
		<0.0001	0.86	0.29
E	NBud	25.4%	1.6%	2.8%
		0.0005	0.82	0.66
F	Total DNA damage	43.1%	1.0%	1.65%
		<0.0001	0.87	0.8

Chromosomal instability (CIN) following 12 days culture in medium containing low (25 nM) or high (100 nM) folic acid (FA), in combination with 0 nM, 400 nM, 1000 nM or 3500 nM cortisol. Frequency of binucleated (BN) cells (per 500 BN) displaying one or more biomarker of CIN; (A) fused (FUS) nuclei; (B) nucleoplasmic bridge (NPB); (C) circular (CIR) nuclei; (D) micronuclei (MN); and (E) nuclear bud (NBud). (F) Total DNA damage (combined total frequencies of BN cells displaying FUS, NPB, CIR, MN or NBud).

Data represents analysis by two-way ANOVA; duplicate slides scored for N = 6 participants; var, % variance explained by each factor.

For total DNA damage observed (i.e. the combined total frequencies of BN cells displaying FUS, NPB, CIR, MN or NBud), ANOVA analysis indicated that [cort] explained only 1% of the variance (p = 0.86, ns). The effect of [FA] was strongly significant, explaining 43% of variance (p < 0.0001), whereas only 1.6% was attributable to the interaction of FA and cort (p = 0.7, ns) (Table 2).

In the case of FUS morphologies, in FA(low) cultures cort exposure resulted in increasing frequencies of FUS, whereas in the FA(high) cultures cort had the opposite effect, with decreasing FUS observed (interaction p = 0.24, ns) (Fig. 2A). Cort exposure had an apparent protective effect against NPB with decreasing frequencies observed in the cultures containing cort. In these cultures the interaction of FA and cort explained 11.5% of variance (p = 0.07, ns) (Fig. 2B).

### Cell viability, growth and cytostasis

Indicators of cytostasis were measured to determine the effect of cort and FA, individually and interactively, on cell viability, apoptosis, necrosis, and cell division.

FA had a highly significant effect on cell viability, explaining 57% of variance (ANOVA, p < 0.0001), while the effect of cort was negligible (1.7% of variance, p = 0.63). Mean viability for FA(low) cultures at day 12 (64% ± 3) was 30% lower than that of FA(high) cultures (84% ± 1). In the FA(low) cultures viability of the three cort-containing conditions were consistently 5–10% higher than that of VC cultures, however, this difference was not statistically significant (p = 0.6).

At day 12 [FA] explained 37% of variance in the frequency of apoptotic, and 45% of necrotic cells (both p < 0.0001), while only 2% (p = 0.6) and 4% (p = 0.3), of variance, respectively, was attributable to [cort].

**Fig 3:**
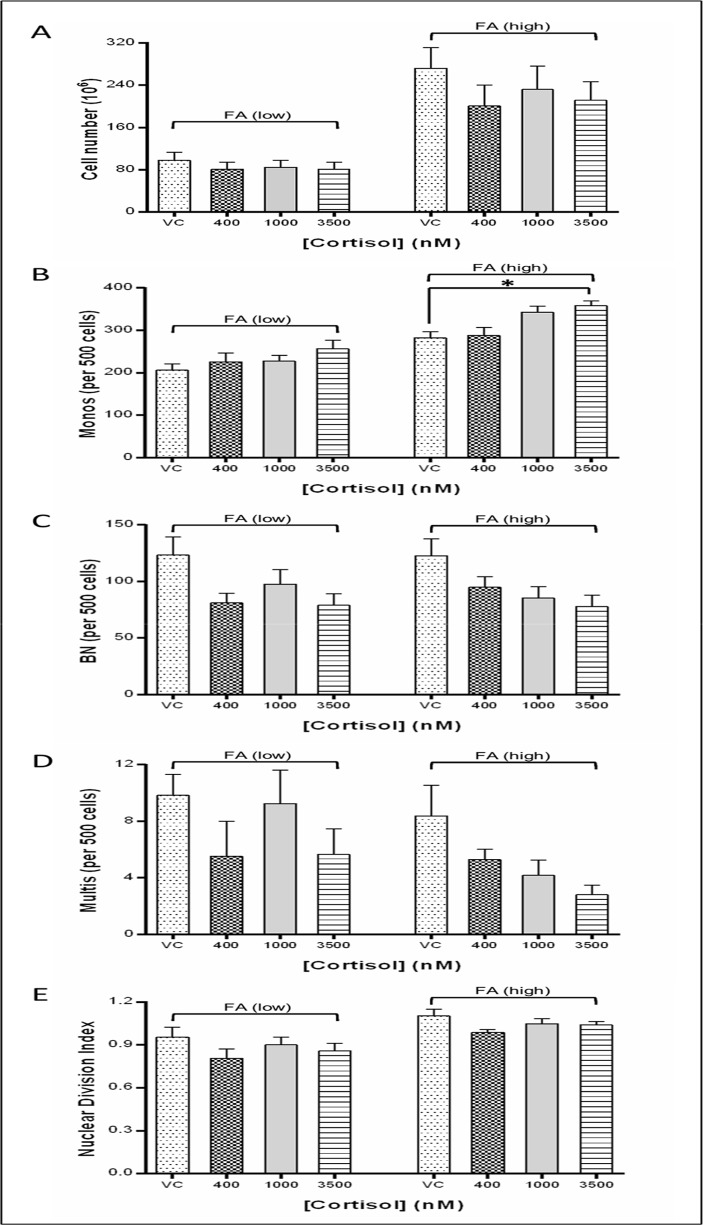
Impact of cortisol on lymphocyte cell division following 12 days in low folic acid (FA) (25 nM) or high FA (100 nM) culture conditions. (A) Cell growth. (B-D) frequencies (per 500 total cells) of mononucleated cells (monos); (C) binucleated cells (BN); and (D) multinucleated cells (mulits). (E) Nuclear division index (NDI); (duplicate measures for N = 6 participants. Mean ± SEM. *p ≤ 0.05).

The mean total number of viable cells recorded at day 12 was significantly higher in FA(high) (229 ± 31 x 10^6^) compared with FA(low) cultures (86 ± 8 x 10^6^) (p < 0.0001). In cort-containing cultures viable cell counts were 16–36% lower than the respective cort-free control (VC) (ns). At day 12 [FA], [cort] and their interaction were responsible for 53% (p < 0.0001), 3% (p = 0.4) and 2% (p = 0.5) of variance, respectively (Fig. 3A).

With regard cell division, at day 12 [cort] significantly impacted the frequencies (per 500 cells) of mononucleated cells (monos) (explaining 12% of variance, p = 0.005), binucleated cells (BN) (28%, p = 0.003) and multinucleated cells (multis) (16%, p = 0.05) (Fig. 3B-D). [FA] was responsible for 50% (p < 0.0001), 4% (p = 0.9) and 7% (p = 0.05) of variance in monos, BN and multis, respectively. When calculated as a single nuclear division index (NDI) value 17% of variance was attributable to [FA] (p = 0.004) and 7.5% to [cort] (p = 0.27, ns) (Fig. 3E).

## Discussion

Since the key work by Epel et al demonstrated that mothers with an autistic child had significantly shorter telomeres than mothers of non-autistic children, many studies have observed a link between exposure to chronic psychological stress and accelerated ageing at the cellular level [6]. Women with the highest perceived (self-reported) stress had lower telomerase activity and shorter leukocyte TL, equivalent to approximately one decade of ageing, compared to those with lower perceived stress [6]. This association has subsequently been shown in carers of chronically ill family members [37, 38], children exposed to violence [39], victims of intimate partner (domestic) violence [40], and individuals with pessimistic or depressive psychological states [41, 42]. Chronic stress has also been associated with compromised immune function, poor response to vaccination, elevated production of inflammatory mediators, and cardiovascular disease risk [37, 38, 43, 44].

Positive lifestyle factors such as consuming a healthy diet, meditation and exercise appear to offer a protective (and in some cases a restorative) effect to telomeres [3, 7, 45]. Folate is required as a co-factor for accurate replication of DNA, maintenance of telomere integrity and DNA methylation, and is essential for protection against chromosomal instability (CIN) [15, 17]. In this study we hypothesised that (i) cortisol induces telomere shortening and CIN, and as such is potentially a mediating factor for the shorter telomeres observed *in vivo* in chronically stressed individuals, and (ii) that susceptibility to CIN and telomere attrition by cortisol is exacerbated when folate is deficient. To test these, the interactive effects of cortisol and FA were examined by comparing telomere length (Q-FISH flow cytometry), biomarkers of DNA damage (CIN), cytostasis (CBMN-cytome assay), cell growth and viability in primary human lymphocytes *in vitro*.

Our findings refuted the hypotheses. Following 12 days of chronic exposure to cortisol *in vitro* lymphocyte telomeres were, in fact, longer than those of cells maintained for the same period in cortisol-free (control) medium. This effect was most evident in the FA-replete conditions. Consistent with previous findings in human WIL2-NS cells, TL was consistently longer in all FA deficient cultures, relative to the FA replete conditions [17]. A known mechanism for telomere elongation is DNA hypomethylation, which occurs when FA is limiting [17, 36]. As such, we considered whether cortisol may have an impact on DNA methylation status. Results showed that the cells with the longest TL were also the most hypomethylated, however, the overall associations between FA deficiency, DNA hypomethylation, and TL in these samples were inconclusive, possibly reflecting different susceptibilities to FA deficiency between different cell types.

Previous findings have shown that, under certain conditions, abnormally elongated telomeres can be as dysfunctional as critically short telomeres, leading to increased CIN and disease risk [17, 46–48]. *In vitro* and animal studies have also suggested that stress hormones may induce damage to DNA [29, 49–51]. Short term (< 30 minutes) exposure to cortisol *in vitro* was shown to disrupt DNA repair in murine 3T3 cells exposed to UV, including transcriptional modulation of 21 genes involved, directly or indirectly, in DNA repair [49]. Accordingly, we incorporated a comprehensive analysis of chromosomal stability and DNA damage using the CBMN-cytome assay to determine whether cortisol is directly genotoxic in primary lymphocytes. This assay has been shown to be sensitive to the DNA damaging and cytotoxic effects of oxidants such as hydrogen peroxide, and superoxide [52]. Consistent with previous findings the effects of FA insufficiency resulted in significant increases in DNA damage. For cortisol, however, with the exception of FUS morphologies, the results were not significant. In the low FA cultures cortisol exposure was associated with a significant increase in fused nuclei (FUS), a non-significant increase in NBuds, no change in the frequency of MN, and a reduction in NPB and CIR. FUS morphologies, which increased with increasing cortisol, may occur due to failure of chromatid separation, suggesting that cortisol may adversely affect mitosis [35].

In FA replete cultures cortisol had no effect on the frequency of MN or NBuds, but a trend for a reduction in FUS and NPB was observed. Previous *in vivo* findings by Malvandi et al showed that restraint stress in mice increased the induction of MN in bone marrow of vinblastine-treated animals, compared with controls [29]. Evidence by York et al also suggested a link between stress exposure and DNA damage, showing an increased frequency of MN in adults who had suffered social abuse in childhood [10]. Findings in the present study suggest that cortisol, in isolation, is not directly genotoxic, in either the high or low FA conditions. This is consistent with the TL data, and further indicates that cortisol is unlikely to be the mediating factor in stress-related genomic instability.

Consistent with previous findings cell division was directly, and negatively, impacted by the presence of cortisol, with a significantly increased frequency of mononucleates, at the expense of bi- and multi-nucleated cells [12, 13]. This effect occurred in parallel with a slight elevation in cell viability in the low FA cultures, possibly suggesting cortisol may stimulate a protective homeostatic mechanism in lymphocytes under conditions of metabolic stress.

The present observations warrant further investigation in a larger sample size, possibly maintaining cells for a longer period in high (physiologically relevant) cortisol conditions, and/or simulating repetitive acute stress and recovery cycles. We are currently undertaking to examine these same biomarkers in the presence of noradrenaline, the stress hormone released in parallel with cortisol upon HPA activation. Under well-controlled conditions, such *in vitro* studies provide useful insights into relationships and mechanistic effects of specific factors. The limitations of these models should, however, be noted with respect to extrapolation to an *in vivo* setting. For example, oxygen tension *in vitro* culture is higher than *in vivo*, and the composition of culture medium differs from that of body fluids, such as blood plasma [53].

### Conclusions

Our findings indicate that cortisol exposure, and folate deficiency, cause telomere elongation in primary lymphocytes, however the effect of cortisol on TL was marginal relative to that of FA deficiency. We further concluded that cortisol is not directly genotoxic, but does negatively impact cell division, a mechanism which may contribute to compromised immunity observed in chronically stressed individuals. Taken together these findings suggest that *in vivo* telomere shortening associated with psychological stress may not be explained by a direct effect of cortisol.
